# Sustainable Hybrid Latexes Derived from Starch Bioparticles and Biobased Monomers

**DOI:** 10.1021/acs.biomac.5c01315

**Published:** 2025-08-29

**Authors:** Sofía F. Cabrera, Aitor Barquero, Ludmila I. Ronco, Sara Beldarrain, Luis M. Gugliotta, Roque J. Minari, Jose R. Leiza

**Affiliations:** † Polymer Reaction Engineering Group, 132107 INTEC, Santa Fe 3000, Argentina; ‡ POLYMAT, Kimika Aplikatua Saila, Kimika Fakultatea, 160665 University of the Basque Country UPV/EHU, Donostia/ San Sebastian 20018, España; § Facultad de Ingeniería Química, Universidad Nacional del Litoral, Santa Fe 3000, Argentina

## Abstract

Starch is a widely available biopolymer with the potential to replace petroleum-based acrylic monomers in coating binders, but its hydrophilicity and water sensitivity limit its incorporation. This study enhances biobased content in hybrid latexes by simultaneously incorporating starch/zein bioparticles (BPs), produced via nanoprecipitation, and commercial biobased monomers including 2-octyl acrylate, 2-octyl methacrylate, and isobornyl methacrylate. Waterborne hybrid dispersions were synthesized through semibatch emulsion polymerization, using BPs as seeds with variable biobased formulations. The influence of solids content, increased from 20% to 32%, was also examined. The resulting hybrid latexes achieved biobased contents exceeding 70% and demonstrated excellent film-forming properties. When applied in paint formulations, they yielded stable coatings with high surface hydrophobicity (contact angle of 98°) and good coverage. These findings demonstrate the potential of combining starch-based bioparticles with bioderived monomers to create sustainable waterborne coatings with high biobased content and competitive performance.

## Graphical abstract

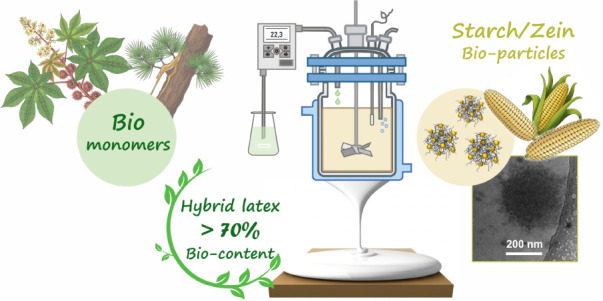

## Introduction

In recent years, the development of more sustainable and environmentally friendly polymer materials has been motivated by the instability of oil prices, consumer demands, environmental concerns, and increasing regulations of greenhouse gas emissions (particularly CO_2_). In light of this, there is a growing interest in reducing the use of petroleum-derived raw materials in favor of renewable alternatives, with the aim of lowering the carbon footprint. In addition, waterborne polymers, such as latexes, serve as an eco-friendly alternative to solvent-based resins in many industrial applications, including coatings, adhesives, packaging, inks, and more, reducing the emission of volatile organic compounds.

In this context, the development of hybrid latexes produced from biopolymers and synthetic polymers represents a promising class of waterborne materials, as it can synergistically combine properties contributed by each component.^1,2^ The incorporation of biopolymers allows for the partial replacement of petroleum-based polymers and could provide other functional properties to the hybrid material, such as film-forming ability, balanced mechanical properties (hardness and blocking resistance), and partial biodegradability.^3–6^

One of the incorporated biopolymers in emulsion polymerization is starch, employing it both as a soluble material^7–9^ and in the form of nanoparticles^10–13^ as a stabilizer. However, only up to 10% incorporation has been achieved. A high degree of starch incorporation has been previously reported, where polysaccharide-based nanoparticles were used as seeds in an emulsion polymerization.^14–18^ These nanoparticles were subjected to chemical modifications, such as cross-linking and surface functionalization with vinyl groups to produce a tie-layer that enhances their stability and hydrophobicity before their use as seeds. Lately, the synthesis of starch-based bioparticles (BPs) has been reported through a nanoprecipitation process in the presence of zein,^19^ a protein obtained as a byproduct of bioethanol and cornmeal production. Zein’s hydrophobicity, combined with starch, allows the optimization of BPs production, enabling their use as seeds in the emulsion polymerization of petroleum-based acrylic monomers, while a second protein, casein, was employed as a biomacroemulsifier.^20^ Subsequently, the incorporation of starch-zein BPs as seeds in an emulsion polymerization was reported, in which a low molecular weight emulsifier (Dowfax 2A1) was used as a stabilizer. The acrylic monomeric composition was optimized, resulting in starch-based hybrid latexes with 20% solids and approximately 20% biocontent, exhibiting promising properties for their use as binders in coatings applications.^21^

Although significant progress has been made in incorporating starch-zein BPs into acrylic hybrid latexes, further improvements are needed to increase both the solids content to levels of coating industry interest and the biocontent of the latexes. However, the increase in BPs concentration to improve the biocontent is mainly limited by the deterioration of the water resistance of hybrid films. A promising approach to increase the biocontent is the replacement of traditional petroleum-based monomers with those derived from renewable sources. Recently, new biobased monomers able to polymerize via free radical mechanisms have been developed, and many of them are commercially available. Biobased monomers are obtained through acrylation or methacrylation of derivatives of vegetable oils and their fatty acids (e.g., soybean acrylated macromonomer, acrylated methyl oleate, acrylated ricinoleic acid, and 2-octyl acrylate: 2OA and 2-octyl methacrylate: 2OMA), pine resin (isobornyl (meth)­acrylate: IBO­(M)­A), glucose (isosorbide monomethacrylate), dextrose (alkyl polyglucoside maleic acid ester: Ecomer), among others. Some of these monomers have been proposed as alternatives to substitute petroleum-based monomers in the synthesis of latexes by emulsion and miniemulsion polymerization,^22–24^ with potential applications as waterborne biobased pressure-sensitive adhesives,^25–27^ alkali-soluble resins,^27^ and coatings.^28,29^ Allasia et al. first reported the synthesis of hybrid latexes based on biopolymer (casein) and two bioderived monomers, IBOMA and 2OA, resulting in materials with good mechanical performance, increased water resistance, and improved biodegradability compared to hybrid materials made with petroleum-based monomers.^30^

In this work, we investigate the enhancement of biocontent in acrylic-starch hybrid latexes through the simultaneous incorporation of biomonomers and starch-zein BPs in a seeded emulsion polymerization process. Different biobased monomeric formulations based on 2OA, 2OMA, and IBOMA were investigated. The study began with a solids content of 20%, which was later increased to 32% solids, enabling their use in coating formulations. The film-forming capability of synthesized high-biocontent hybrid latexes, without coalescing agents that make them suitable for low-VOC coatings, was evaluated. Also, as proof of concept, a pigmented paint formulation using a high-biocontent hybrid latex as a binder was assessed.

## Materials and Methods

### Materials

To obtain the BPs, food-grade native corn starch (generously supplied by Glutal S.A., Argentina) and zein (Sigma-Aldrich) were used. The biobased monomers employed in the synthesis of hybrid latexes were IBOMA (VISIOMER Terra IBOMA) and 2OMA (VISIOMER Terra OCMA), kindly supplied by Evonik, and 2OA, which was synthesized by Fischer esterification, adapting the method reported by Barrenetxe et al.^23^ Additionally, the petroleum-based monomers used were methyl methacrylate (MMA, Quimidroga) and *n*-butyl acrylate (BA, Quimidroga). Potassium persulfate (KPS, Sigma-Aldrich) was employed as an initiator, Dowfax 2A1 (45% aqueous solution, kindly supplied by Dow Chemical) as a surfactant, and sodium carbonate (Na_2_CO_3_, Sigma-Aldrich) as a buffer. Other reagents were tetrahydrofuran (THF, Scharlau) for the insoluble fraction determination, ethanol (96% v/v) for the BPs synthesis, and methyl ethyl ketone (MEK, Anedra) for the solvent resistance. Dispelair CF 246 (mineral oil) was used as a defoamer (kindly provided by Blackburn Chemicals), Bermocoll EHM (methyl ethyl hydroxyethyl cellulose, a water-soluble cellulose ether) as a thickener (kindly provided by Akzonobel Chemicals AG), and Orotan 731A ER (water-soluble polycarboxylate, sodium salt of a carboxylate polyelectrolyte) as a dispersing agent (Dow Chemical). The inorganic pigment Ti-Pure R706 titanium dioxide (TiO_2_) was generously supplied by Chemours. All chemicals were used as supplied, without further purification, and both distilled and deionized water were employed throughout the study.

### Synthesis of Starch/Zein BPs

Starch/zein BPs were produced by the antisolvent precipitation method reported in Cabrera et al.,^20^ which involves precipitating an aqueous starch solution (the “solvent”) into an alcoholic zein solution (the “anti-solvent”). In a typical synthesis, 20 mL of the starch solution (4.4% w/v), previously obtained by gelatinizing starch at 90 °C, and 67 mL of the zein solution (1.5% w/v), prepared by dissolving zein in ethanol (90% v/v), were used. The pH of the starch solution was adjusted to 11 by adding Na_2_CO_3_ (final concentration of 0.4% w/w). To form the BPs, the starch solution was introduced dropwise into the zein solution at a constant flow rate of 1.2 g/min, while continuously stirring and applying sonication (10 s on and 5 s off) with an amplitude of 70% using an ultrasonic processor Sonics VC 750 (750 W). The resulting BPs dispersion was centrifuged at 5000 rpm for 15 min, washed with 70% (v/v) ethanol to eliminate the excess zein, and then centrifuged again under the same conditions. The BPs were then redispersed in water, lyophilized, and stored at −15 °C for later use.

### Emulsion Polymerization

Hybrid latexes were synthesized via seeded semibatch emulsion polymerization using BPs as seeds and acrylic formulations based on the biobased monomers 2OA, IBOMA, and 2OMA. Table S1 provides details on their origin, biobased carbon content, and the *T*_g_ of the corresponding homopolymers.

Two groups of hybrid latexes with different solid contents of 20% and 32%, and BPs/acrylic weight ratios of 25/100 and 11/100, respectively, were produced. In all cases, an aqueous pre-emulsion, containing Dowfax 2A1 as an emulsifier and biobased monomers, was fed at a constant rate. Three formulations based on biomonomers were studied to produce both low solids content (LS) and high solids content (HS) latexes: (i) IBOMA/2OA: 25/75 weight ratio (theoretical *T*_g_ = −14 °C, calculated using the Fox equation); (ii) IBOMA/2OA: 35/65 (theoretical *T*_g_ = 0 °C); and (iii) 2OMA (theoretical *T*_g_ = 0 °C). Additionally, a hybrid latex with a petroleum-based monomeric formulation of BA/MMA: 60/40 (theoretical *T*_g_ = −10 °C) and 20% solid content was synthesized under the same conditions. It is interesting to highlight that acrylic formulations with higher *T*_g_ values are typically used as binders in coating applications to enhance mechanical properties and blocking resistance. However, we have previously demonstrated that acrylic-BPs hybrid latexes with subzero *T*_g_ are also suitable for coatings owing to the functional properties provided by BPs.^21^ Table 1 presents the typical recipe used in the emulsion polymerizations of the two groups of latexes with 20–32% target solids content.

**Table 1:** Typical Polymerization Recipe Employed in the Synthesis of Hybrid Latexes

Reagent/experiments		20%-LS (%wt)	32%-HS (%wt)
Initial charge	BPs	4.05	3.35
	Water	68.48	47.60
Pre-emulsion	Biobased monomers	16.22	30.15
	Dowfax 2A1	0.45	0.74
	Water	6.94	12.92
Initiator solution	KPS	0.26	0.48
	Water	3.60	4.76
Total		100.00	100.00

A 100 mL jacketed reactor was employed, equipped with an inlet for nitrogen, a sampling port, a condenser, and mechanical stirring. To control the reaction temperature, a thermostat bath connected to a reactor jacket was used. Initially, BPs were redispersed in water by magnetic stirring for 2 min, followed by sonication at 50% amplitude for 30 s. The BPs dispersion was loaded into the reactor and purged with nitrogen under constant mechanical stirring. The reaction temperature was maintained at 60 °C. Then, the initiator solution (KPS in 3 mL of water) was added as a shot to start the polymerization, and simultaneously the feeding of the pre-emulsion was initiated. In both reaction groups, the same constant feed rate was used, involving a feed time of 3 h for latexes synthesis with 20% solid content and 6 h in the case of latexes with 32% solids content. In all cases, the conditions were maintained for an additional hour after the end of the dosing time for postpolymerization. Samples were taken at regular intervals during the polymerization to measure monomer conversion (*x*) and the average particle diameter (*d*_*p*_).

### Characterization of Hybrid Latexes and Films

The overall conversion was measured gravimetrically. The intensity-based *d*_*p*_ and polydispersity index (PDI) were determined using dynamic light scattering (DLS) with a Nanosizer (Malvern) at 25 °C. An estimation of the particle number (*N*_*p*_) was calculated from the results of *x* and *d*_*p*_, assuming spherical and monodisperse particles.

The morphology of BPs was studied by cryo-TEM. For this, BPs were dispersed in water at a concentration of 0.25 mg/mL, and this dispersion was washed to remove some soluble starch through centrifugation and redispersion of the precipitate in distilled water. Quantifoil R2/1 grids were used, which were pretreated with glow discharge (air plasma) for 2.5 min. A volume of 3 μL of the cleaned BPs dispersion was placed onto the grid and vitrified using a ThermoFisher Vitrobot Mark IV system in liquid ethane. Images were acquired on a JEOL 1400 Plus microscope at 80 kV and equipped with a LaB_6_ electron source. In addition, TEM was used to examine the morphology of hybrid latex particles with a JEOL JEM-2100 Plus microscope operated at 200 kV. The samples were diluted to approximately 0.1 wt % solid content in deionized water, and a droplet of the dispersion was deposited onto a carbon-coated copper grid, followed by drying at room temperature. Micrographs were acquired at various magnifications.

The minimum film formation temperature (MFFT) was measured in Rhopoint-Model 90 equipment. For this, the latex was applied to a steel plate using a 120 μm wet-thickness quadrangular applicator, with the plate subjected to a temperature gradient ranging from −5 to 10 °C. MFFT was defined as the temperature at which the film became optically clear and showed no cracks.

Hybrid films were obtained by casting the latexes onto silicone molds at 23 ± 2 °C and 55 ± 5% relative humidity until ensuring constant film weight (about 7 days), yielding a final thickness of approximately 1 mm.

The acrylic insoluble fraction (AIF), representing the portion of the acrylic copolymer that is insoluble in a good solvent, was determined via Soxhlet extraction for 24 h using THF, which dissolves the soluble acrylic polymer but does not dissolve the biopolymers. The AIF was calculated by subtracting the biopolymer mass from the total mass of the film sample.

The film’s morphology was analyzed by TEM. For this, an ultracryomicrotome (LEICA EM UC) with a cryochamber (LEICA EM FC6) was used to obtain cross-sectional slides of film samples, each 100 nm thick. Film slides were collected on a copper grid and observed with a TECNAI G2 20 TWIN (200 kV, LaB6) microscope.

Differential Scanning Calorimetry (DSC) was conducted using a Q2000 instrument from TA Instruments. Film samples weighing 3–5 mg were analyzed in a sealed aluminum pan (TZero technology) with a heating rate of 10 °C/min from −90 to 130 °C.

Water contact angle (CA) on the film surface was measured using a Contact Angle System OCA (Dataphysics) equipment. Hybrid latexes were cast on glass slides with a 120-μm-thick frame applicator and dried at room temperature for 24 h. Water droplets (25 μL) were placed on the film surface, and the CA was measured under equilibrium conditions. The average CA from 20 measurements was reported for each hybrid film.

Water and solvent resistances were tested on film samples (5 × 5 mm, 0.5 mm thick) immersed in water and methyl ethyl ketone (MEK) at room temperature. At regular intervals, samples were removed, dried with a paper filter, weighed, and reimmersed. The relative mass of solvent (water or MEK) absorbed and the weight fraction of film loss after 7 days of water immersion were calculated.

Film opacity was measured by determining the absorbance spectrum (400–800 nm) of films (∼0.5 mm thick) using a UV–vis spectrophotometer (PerkinElmer, lambda 25). Opacity was defined as the area under the absorbance spectrum divided by the film thickness.

Tensile tests were conducted using a universal testing machine (INSTRON 3344) at 25 °C, 50% relative humidity, and an elongation rate of 25 mm/min on dumbbell-shaped specimens with an elongation section of 26 mm in length, 3 mm in width, and 0.8 mm in thickness. At least three specimens from each sample were tested.

### Paint Formulation and Characterizations

A paint was formulated by first preparing a mill base, where deionized water, defoamer, cellulose-based thickener, and dispersing agent were mixed before the addition of TiO_2_ as an inorganic pigment. The mixture was subjected to high shear forces of the dissolver used for milling the inorganic particles. The milling was carried out for 20 min at 1000 rpm. Then, the mill base was filtered using a 190 μm pore-size paint strainer, and it was left overnight before its addition to the let-down. The let-down was prepared by mixing the hybrid latex with the mill base and some defoamer, and mixing it for 20 min at 500 rpm using a straight-blade impeller as a stirrer. Table 2 shows the formulation used.

**Table 2:** Paint Formulation Recipe for a 23% Pigment Volume Concentration (PVC) and 35% Solids Content Waterborne Paint^a^

Mill base	wt %
Water	51.4
Dispelair CF 246^a^	1.2
Bermocoll EHM extra	0.6
Orotan 731A ER^b^	5.6
Ti-Pure R706	41.2
Mill base total	100.0

Let-down	wt %
Hybrid latex	49.5
Mill base	49.5
Defoamer^a^	1.0
Paint total	100.0

a 99% active solution.

b 25% active solution.

The paint obtained was applied to different substrates for its characterization. Gloss, surface roughness, opacity, contact angle, and water resistance were analyzed. The water contact angle and water resistance were measured in the same way as reported above for hybrid films. The gloss was measured on a film applied at 200 μm wet thickness using a gloss meter (micro-TRI-gloss, BYK). The gloss was measured with the 85° detector due to the matte appearance of the coating (<10 GU when measuring with the 60° detector).^31^ The surface roughness was tested on a film applied over a glass substrate at a 200 μm wet thickness by using a surface roughness tester (PCE-RT 10, PCE). The equipment is composed of a diamond-tipped sensor that uniformly scans the surface, detecting the peaks and valleys of the surface. The reported value (Ra) is the arithmetic mean deviation of the profile within a sampling length. Opacity was measured by applying the paint at different thicknesses on a white and black substrate (reference standard) and using an opacity meter (Novo-Shade Duo reflectometer, Rhopoint instruments).

## Results and Discussion

### Synthesis of Hybrid Latexes

In this work, we investigate the synthesis of waterborne hybrid starch-based latexes with high biocontent through semibatch emulsion polymerization of biobased monomers, using starch-zein BPs as seed (Figure 1A).

**Figure 1:**
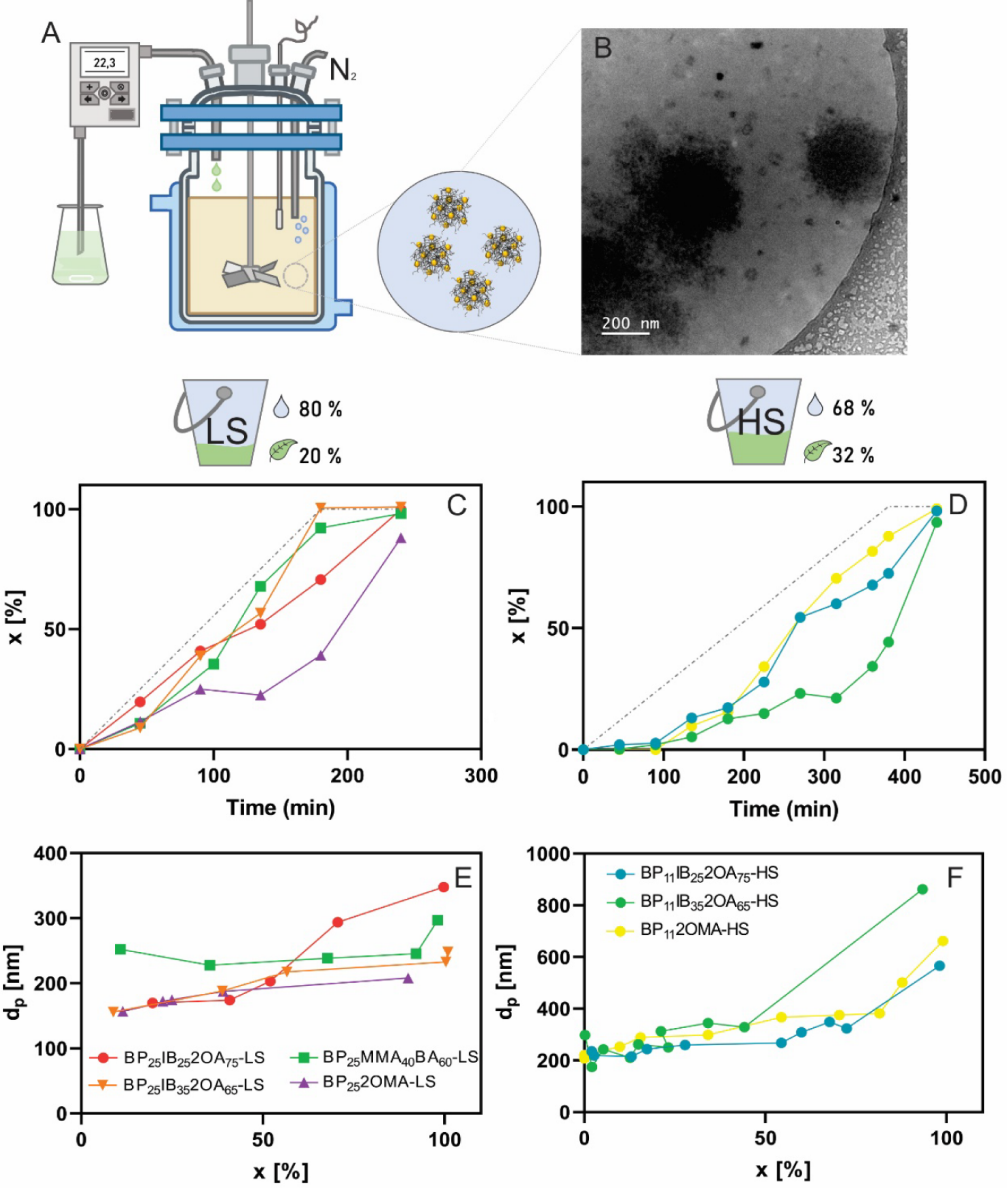
Scheme of the synthesis of hybrid latexes by semibatch emulsion polymerization, using starch/zein-based BPs as seed and feeding a pre-emulsion of biobased monomers (A). Cryo-TEM image of BPs (B). Evolution of *x* (C, D) and *d*_*p*_ (E, F) along the polymerization for the synthesized latexes with 20% (C, E) and 32% (D, F) of theoretical solids content.

Starch/zein BPs were prepared by nanoprecipitation, adding a water solution of starch (the “solvent”) into an ethanol solution of zein (the “anti-solvent”), causing the starch precipitation and its physical cross-linking with zein. The complexation process involves the formation of hydrogen-bonding interactions between the amino (carboxyl) groups of the protein and the hydroxyl groups of starch.^32,33^ This method allows the production of starch-based BPs with around 4% zein, which acts as a physical cross-linker uniformly distributed within the bioparticle.^20^

The Cryo-TEM image in Figure 1B shows the appearance of BPs in the dispersion. Large BPs, exceeding 200 nm in size, can be observed, in addition with small BPs with sizes under 50 nm. Both populations exhibit a nondense structure, which is attributed to the swelling properties of starch in water. Moreover, the average *d*_*p*_ of BPs measured by DLS at a low concentration (0.25 mg/mL) was 281 nm, which agrees with the cryo-TEM observations, considering that the DLS measurements are primarily influenced by the larger particles. In addition, BPs tend to form aggregates, a process influenced by the increasing concentration of BPs, as previously demonstrated.^20^ Therefore, the use of BPs dispersion as a seed involves a dynamic system composed of large and small BPs, dominated by BP aggregates, which can undergo changes in both size and quantity during the polymerization process.

A first group of latexes was synthesized with a solids content of 20% and a BPs/monomer weight ratio of 25/100. Moreover, the synthesis of hybrid latexes with a higher solids content (32%) was investigated, employing the same biobased monomeric formulations. In these cases, a BPs/monomer ratio of 11/100 was selected, as using a ratio of 25/100 at high solids content led to latex destabilization during polymerization (Figure S1). The latex destabilization was likely caused by the significant increase in the viscosity of the initial seed dispersion due to the higher concentration of BPs. Thus, the BPs/monomer ratio was reduced to achieve a similar BPs concentration in the seed dispersion as in the polymerization with 20% solids content, enabling the production of stable latexes at higher solids contents.

The experiment codes adopted specify the BPs content, followed by the monomeric formulation, and ending with the solids content (LS for 20% solids and HS for 32% solids). For example, the experiment BP_25_IB_25_2OA_75_-LS corresponds to a polymerization carried out with a BPs/acrylic mass ratio of 25/100 (BP_25_), a monomeric formulation of IBOMA/2OA: 25/75 (IB_25_2OA_75_) and 20% solids content (LS).

Table 3 summarizes the final results of all polymerizations that were carried out, while Figure 1 C–F presents the evolution of monomer conversion and *d*_*p*_ along the reactions. For each latex, the total biocontent was calculated considering the biocontent of each monomer (Table S1) and the inclusion of BPs, which are entirely derived from natural sources. The resulting biocontent values range from 70% to 78%, significantly higher than the 20% observed for the analogous hybrid latex produced using the petroleum-derived BA and MMA monomers^20,21^ and even greater than the corresponding homologous latex based solely on these biobased monomers.

**Table 3:** Final Result of Bio-Content, *d*_*p*_, Monomer Conversion, *T*_g_ and AIF for the Synthesized Latexes

Latex	Bio [%]^a^	*d*_*p*_ [nm]/PDI	*x* [%]	*T*_g1_ [°C]^b^	*T*_g2_ [°C]^b^	AIF [%]^c^
BP_25_MMA_40_BA_60_-LS	20.0	296/0.22	98	–10.1	---	49.2
BP_25_IB_25_2OA_75_-LS	78.0	347/0.16	99	–4.7	72.5	45.3
BP_25_IB_35_2OA_65_-LS	77.8	248/0.15	99	11.5	61.3	45.1
BP_25_2OMA-LS	73.6	208/0.13	90	5.0	59.3	17.4
BP_11_IB_25_2OA_75_-HS	75.2	862/0.29	93	–1.4	54.0	36.8
BP_11_IB_35_2OA_65_-HS	75.1	566/0.66	98	10.7	59.8	31.9
BP_11_2OMA-HS	70.3	662/0.29	99	7.4	63.7	26.1

a Calculated from the theoretical formulation, considering the amount of BPs and the biobased content of the biomonomers.

b Calculated from the first heating cycle.

c Fraction of the acrylic copolymer that is insoluble in THF.

The semibatch polymerization of a biobased monomeric formulation with 20% solids gives a conversion profile similar to that of the experiment with conventional petroleum-based monomers (BP_25_MMA_40_BA_60_-LS). In addition, the evolution of conversion for both the LS (Figure 1C) and HS formulations (Figure 1D) remained below the dosing profile (dashed lines), indicating that the polymerization proceeded at a lower rate than the monomer feed rate but reached final conversion values above 90% in all synthesized latexes (Table 3). For this reason, a drift in the copolymer composition could be expected during polymerization due to differences in the comonomer reactivities (*r*_IBOMA_ = 1.609; *r*_2OA_ = 0.428, as predicted by refs 34,35 ).

The average *d*_p_ increased with monomer conversion (Figure 1E,F), reaching final values ranging from 208 to 347 nm for the LS formulations and from 662 to 862 nm for the HS formulations. Moreover, the PDI values were higher than 0.1 for all latexes, suggesting broad particle size distributions (Table 3). In the case of HS polymerizations, until almost 70–80% of monomer conversion, the values of *d*_p_ are below 400 nm; however, significantly larger *d*_*p*_ were observed toward the end of the polymerization (Figure 1F). This phenomenon could be explained by the aggregation of latex particles, driven by the higher solid content, despite none of the latexes exhibiting visible coagulum formation. For the LS latexes BP_25_IB_25_2OA_75_-LS and BP_25_-MMA_40_BA_60_-LS, a sudden change in *d*_p_ at high conversion can also be observed, likely caused by the aggregation of low *T*_g_ latex particles (Figure 1E).

We previously reported that during the synthesis of hybrid latex by semibatch polymerization using BPs as seeds and conventional monomers BA and MMA, a high number of particles are generated through secondary nucleation mechanisms, such as homogeneous and micellar nucleation, in addition to the particles formed via seed-mediated polymerization.^21^ A similar behavior in the nucleation process occurred with the biobased monomeric formulations studied here, as evidenced by the growth of *N*_p_ primarily at the early stages of polymerization (Figure S2). It is hypothesized that the secondary nucleation mechanism corresponds to micellar nucleation, promoted by the incorporation of Dowfax 2A1 in the pre-emulsion dosing. It is worth noting that the concentration of Dowfax used exceeded the corresponding critical micellar concentration in the polymerization system after a few minutes of pre-emulsion dosing.

Figure S3 shows the derivative DSC thermograms of dried hybrid latexes. Two glass transitions are detected, corresponding to the acrylic copolymer (*T*_g1_) and starch (*T*_g2_), respectively. *T*_g2_ values were consistent with those previously reported for starch in acrylic-starch hybrid materials,^20^ meanwhile *T*_g1_ is in agreement with the *T*_g_ expected according to the biobased monomeric formulation (Table 3). These DSC results confirm the presence of two phases in the hybrid materials: the acrylic phase and the BPs phase. However, it is observed that *T*_g1_ is several degrees higher than the values predicted by the Fox equation for the pure acrylic copolymer of IBOMA and 2OA, i.e., −14 °C and 0 °C for formulations IB_25_2OA_75_ and IB_35_2OA_65_, respectively, as well as for poly­(2OMA) (*T*_g_ = 0 °C, Table S1). This difference could be a consequence of both the broader copolymer composition resulting from incomplete monomer conversion during the dosing period and the presence of high *T*_g_ BPs, which may restrict the mobility of the acrylic phase, acting as a physical cross-linker.

All synthesized hybrid latexes exhibit a moderate AIF content, with values ranging from 17% to 49%. AIF consists of the pure acrylic gel and the acrylic fraction incorporated into the poly­(acrylic graft biopolymer). Consequently, the formation of graft copolymers, along with the occurrence of chain transfer reactions to the acrylic polymer leading to high molecular weight and cross-linked chains, contributes to the higher AIF content. In the comparison of different biobased monomeric formulations with the same BPs content, the AIF was higher when using IBOMA/2OA than with 2OMA, likely due to the higher reactivity for hydrogen abstraction of the acrylate units from 2OA.^36^ In addition, HS polymerization BP_11_IB_25_2OA_75_-HS and BP_11_–IB_35_2OA_65_-HS exhibited AIF values lower than those of their LS counterparts, probably due to the lower monomer conversion during a significant portion of the polymerization, which reduced the likelihood of chain transfer reactions to the polymer. Moreover, the biobased formulation BP_25_IB_25_2OA_75_-LS and its petroleum-based counterpart BP_25_MMA_40_BA_60_-LS, which share a similar acrylic *T*_g_, exhibited comparable AIF values.

### Characterization of Biobased Hybrid Films

Figure 2 shows the characterization results of biobased films obtained from LS hybrid latexes, compared to the hybrid film derived from a petroleum-based acrylic formulation; and Figure 3 presents the results of biobased hybrid films prepared from HS latexes.

**Figure 2:**
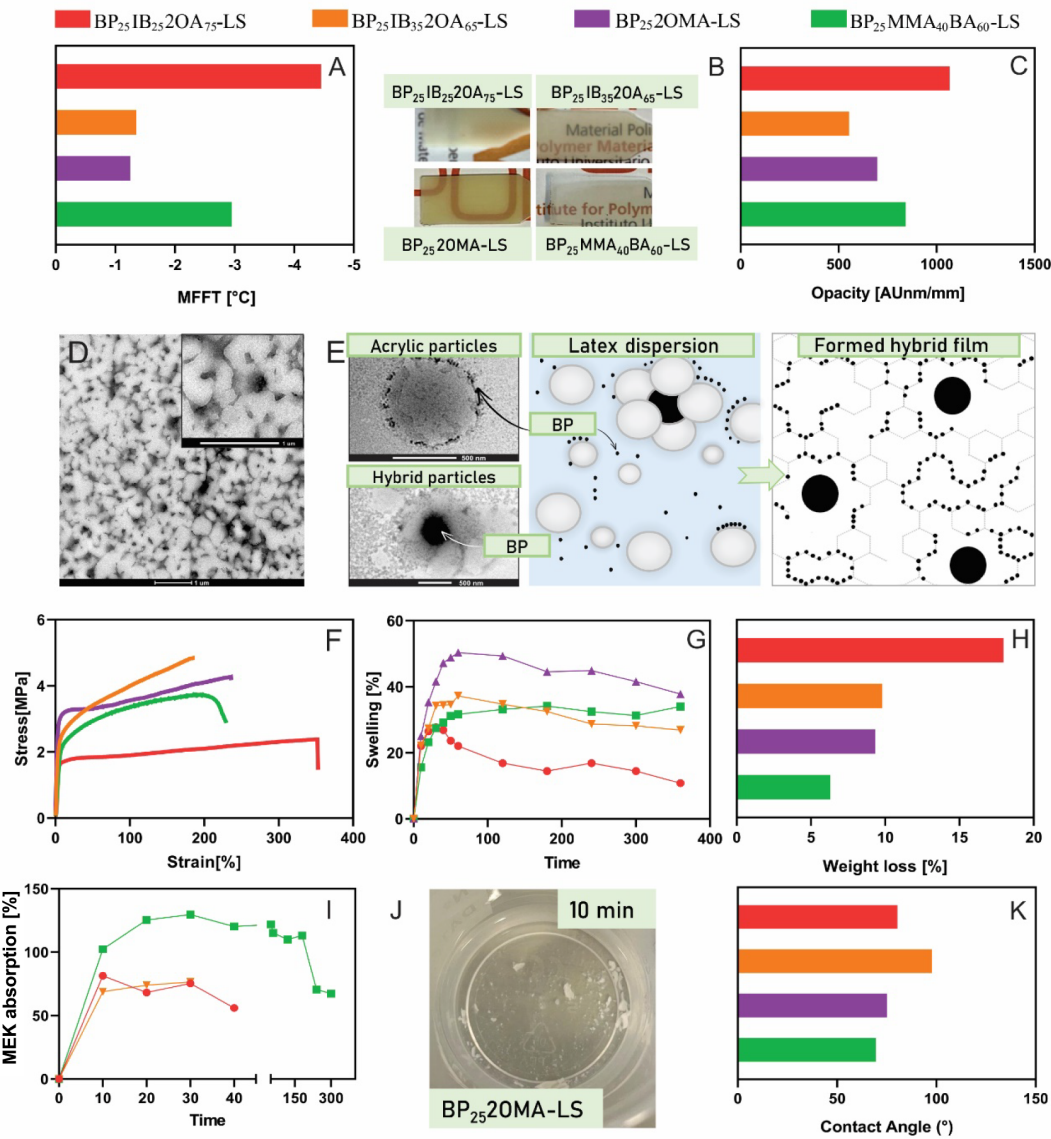
Properties of films obtained from the LS hybrid latexes. MFFT (A), film pictures (B), opacity (C), TEM image from cross-section cut of film BP_25_2OMA-LS (D), scheme of both latex morphologies, including TEM picture of a pure acrylic particle and a hybrid particle of latex BP_25_2OMA-LS, and film formed (E), tensile stress–strain curves (F), water absorption during the first 6 h of immersion (G), weight loss after 7 days of water immersion (H), MEK absorption (I), image of BP_25_2OMA-LS film after 10 min of immersion in MEK (J) and CA (K).

**Figure 3:**
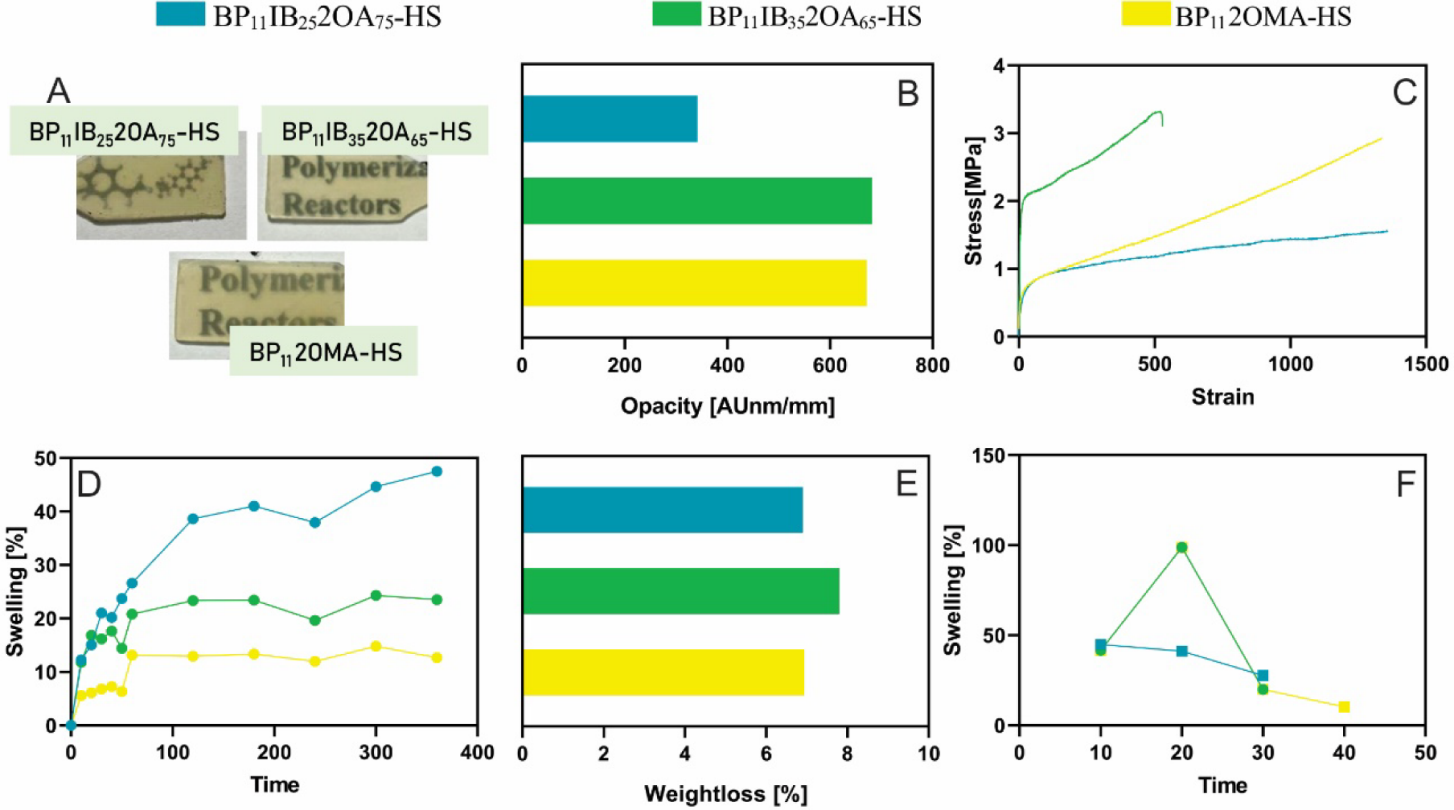
Properties of films obtained from the HS hybrid latexes. Images of the films (A), opacity (B), tensile stress–strain curves (C), water absorption during the first 6 h of immersion (D), weight loss after 7 days of water immersion (E), and MEK absorption (F).

In order to obtain information about the film-forming capability of hybrid latexes, MFFT was measured. All hybrid latexes present MFFT values below 0 °C (Figure 2A) with differences between the formulations that are in agreement with the acrylic *T*_g_. While BP_25_IB_25_2OA_75_-LS has an MFFT of around −4 °C, comparable to the petroleum-based acrylic formulation, synthesized latexes with higher *T*_g_, BP_25_IB_35_2OA_65_-LS and BP_25_-2OMA-LS, form films at temperatures above −1 °C. Moreover, it is worth noting that the MFFT values of samples BP_25_IB_35_2OA_65_–LS (−1.4 °C) and BP_25_2OMA-LS (−1.3 °C) are below the *T*_g_ of their respective acrylic phases. This suggests that hydroplasticization of the starch phase (i.e., BPs and soluble starch) contributes to the film formation process by reducing MFFT.^37^

In addition, homogeneous and translucent films were obtained at room temperature (Figures 2B and 3A), demonstrating the absence of significant phase segregation during film formation. The opacity was evaluated through absorbance measurements, revealing that the BP_25_IB_25_2OA_75_-LS film exhibited the highest value (Figure 2C), consistent with the visual observations (Figure 2B). However, the film with the same biobased formulation, but formed from the HS latex (BP_11_IB_25_2OA_75_-HS), exhibited reduced opacity. In general, when comparing films produced from latexes of LS and HS, the opacity was lower in the latter due to the reduced content of BPs (Figures 2C and 3B). Moreover, these biobased films exhibit opacity comparable to that of the reference MMA/BA formulation.

Figures 2D and S4 show the film morphology observed by TEM from transversal cuts of hybrid films formed from LS latexes. The presence of both phases, acrylic and BPs, can be distinguished inside the films, indicating that the particles have not completely coalesced. To provide further insight into the film morphology, Figure 2E schematizes the composition of polymeric particles in starch-acrylic-based latexes, previously described for acrylic-starch hybrid systems,^20,21^ including a TEM picture of the existing particles from BP_25_2OMA-LS latex. Briefly, two distinct particle populations can be identified: multilobular hybrid particles, consisting of a BPs core (appearing darker) surrounded by acrylic lobes (formed from BPs seed), and smaller pure acrylic particles produced by secondary nucleation. Additionally, small starch-based BPs, which are absorbed onto the surface of the polymeric particles, are also observed. Considering that the *T*_g_ of the acrylic phase is much lower than room temperature, complete coalescence would be expected. However, the presence of small BPs adsorbed onto the polymeric particle surface prevents full polymer interdiffusion. Consequently, the film morphology reveals that the acrylic phase does not fully coalesce; instead, small regions formed by the coalescence of both pure acrylic particles and acrylic lobes of hybrid particles are observed, limited by both large and small BPs. To aid in visualizing the film composition and formation, Figure 2E includes a scheme of the formed film based on the composition of hybrid starch-acrylic latexes. Additionally, the size of the coalesced acrylic phases observed in the film increases as the *T*_g_ decreases, as seen when comparing BP_25_IB_25_2OA_75_-LS and BP_25_IB_35_2OA_65_-LS (Figure S4).

Figures 2F and 3C show the stress–strain curves for hybrid films obtained from LS and HS latexes, respectively, while Tables S2 and S3 provide the tensile mechanical properties such as Young’s modulus, elongation at break, tensile strength, and toughness. Films produced from LS latexes exhibited a higher Young’s modulus and tensile strength but lower elongation at break compared to analogous films obtained from HS (Figure 3C) latexes with the same acrylic formulation. We previously demonstrated that incorporating BPs to produce hybrid materials enhances the mechanical properties compared to pure acrylic films.^21^ This improvement is attributed to the reinforcing effect of hard BP inclusions with a high *T*_g_, embedded in a low-*T*_g_ acrylic phase, which allows significant deformability. Therefore, the differences in mechanical properties of films from LS and HS latexes are mainly associated with the content of BPs, which is higher for LS latexes.

When comparing films derived from IBOMA/2OA biobased monomers, within each group of experiments (LS or HS latexes), the films with higher acrylic *T*_g_ (i.e., BP_25_IB_35_2OA_65_-LS and BP_11_IB_35_2OA_65_-HS) exhibited greater Young’s modulus and tensile strength (Figures 2F and 3C). Moreover, the tensile behavior of these biobased hybrid films is comparable to that of the film derived from MMA/BA monomers (BP_25_MMA_40_BA_60_-LS). However, the tensile properties of MMA/BA film are slightly superior (i.e., higher Young’s modulus and tensile strength) to those of the film with the analogous IBOMA/2OA formulation (BP_25_IB_25_2OA_75_-LS), in terms of a similar *T*_g_ (Figure 2F). Interestingly, all hybrid films showed good deformability and toughness, even though the presence of BPs limits the complete coalescence of the acrylic phase. Moreover, this good mechanical performance could be attributed to the uniform distribution of BPs in the hybrid films (Figures 2D and S4), resulting from the compatibilization of BPs with the acrylic phase on the nanometric scale through the formation of hybrid particles containing both phases during latex synthesis. It was previously demonstrated that the favorable performance of hybrid materials cannot be attained by merely blending acrylic latex with BPs, as the poor compatibility between these components compromises the overall film properties.^21^

Water susceptibility could be a key characteristic to analyze in hybrid films incorporating BPs, as these films contain hydrophilic biopolymers like starch. The swelling results after 6 h are presented in Figures 2G and 3D for films obtained from LS and HS latexes, respectively, highlighting that all materials withstood immersion for 7 days without disintegrating. These biobased films exhibited swelling values below 50%, consistent with the reference BP_25_MMA_40_BA_60_-LS formulation (Figure 2G) and previous results for other hybrid films based on BPs and petroleum-derived formulations.^21^ However, all biobased films lost a higher fraction of their initial weight after 7 days of immersion (Figure 2H). The weight loss likely corresponds to the hydrophilic components, primarily starch, in the BPs. This is why films obtained from HS latexes, with a lower content of BPs, showed reduced weight losses (Figure 3E). Moreover, the film BP_25_IB_25_2OA_75_-LS presented the highest weight loss, which is consistent with its reduced swelling.

During the test of immersion in MEK to analyze the film’s resistance to organic solvents, samples presented an initial high swelling, followed by an abrupt decrease due to their disintegration (Figures 2I and 3F). These results are consistent with those expected for thermoplastic films formed by an acrylic continuous phase, which is susceptible to dissolution in organic solvents. In addition, Figure 2J shows that the BP_25_2OMA-LS film undergoes faster and almost complete disintegration after 10 min of MEK immersion, probably due to the methacrylic nature of 2OMA that yields latexes with a reduced content of AIF. On the other hand, the reference MMA/BA film resisted immersion for a longer time but also disintegrated completely after 300 min.

The CA of hybrid films with water, commonly used as a measure of the hydrophobicity of a film surface,^38^ was determined. The results, shown in Figure 2K, reveal that hybrid films derived from biobased formulations exhibited a higher CA than those from the reference MMA/BA formulation. This increase is attributed to the more hydrophobic nature of the biobased monomers. Similar results were observed in hybrid films produced using casein and IBOMA/2OA monomers.^30^

Based on previous results, the latex BP_11_IB_35_2OA_65_-HS with 32% solids content was selected to evaluate its potential as a binder for hybrid starch-based latexes in formulating a white water-based paint containing 23% PVC and 35% solids content. Figure 4 summarizes the main characteristics of the resulting paint. The surface roughness (Ra) of the resulting paint film was 0.984 ± 0.20 μm, indicating that the pigmented paint formulated with the hybrid starch-based latex formed smooth films. This value falls within the expected range for paints formulated with acrylic binders, such as those based on MMA/BA/acrylic acid, which exhibit a Ra of approximately 1.5 μm at 15% PVC and 41% solids content.^39^ The formulated paint showed a contact angle of 98 ± 5°, which is in agreement with that obtained for the clear film with a similar biobased acrylic composition (BP_25_IB_35_2OA_65_-LS, Figure 2K). To assess the water resistance development over time of formulated paints, Figure 4A shows pictures of water drops after 10 and 20 min of contact with the paint film. At 10 min, the film shows clear deformation upon contact, while after 20 min, initial signs of film damage became evident. This observation is consistent with the weight loss observed for the binder (Figure 3E).

**Figure 4:**
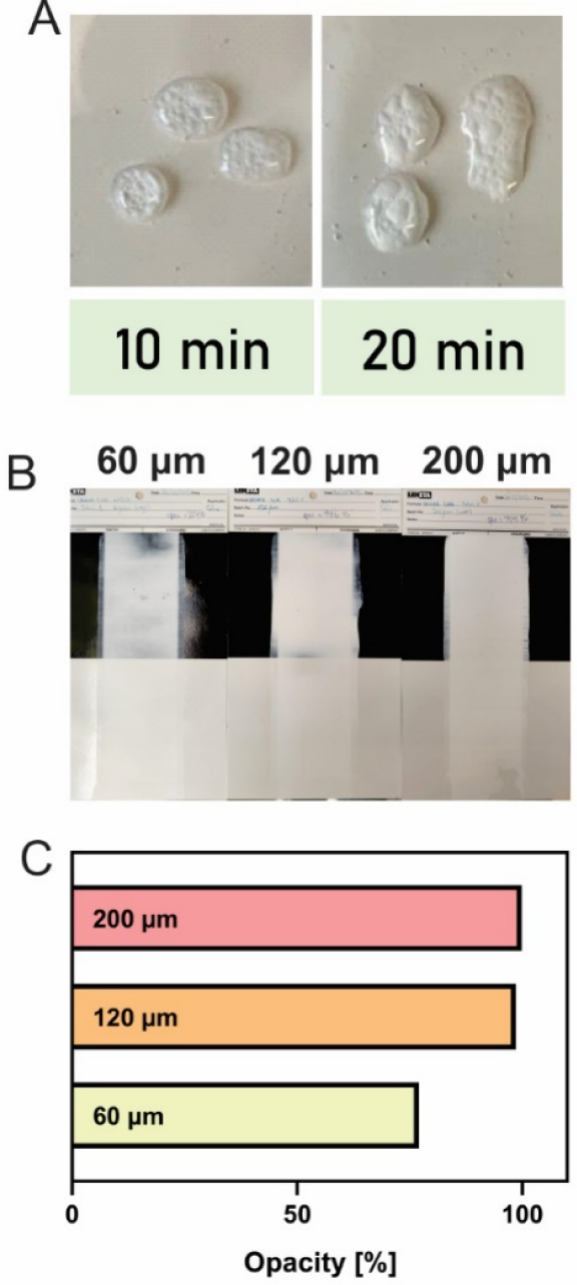
Pigmented paint formulated with BP_25_IB_35_2OA_65_-HS as the binder. Images of water resistance after 10 and 20 min of drop exposure (A), images of the paint applied to reference papers with wet thicknesses of 60, 120, and 200 μm (B), and measured opacity as a function of wet thickness (C).

The gloss was measured using the 85° detector, obtaining a value of 43 GU, which indicates a matte finish for the paint. A visual assessment of hiding power for the formulated paint at wet thicknesses of 200, 120, and 60 μm is shown in Figure 4B, demonstrating good uniformity and coverage at 120 μm and above. As can be observed in Figure 4C, a progressive increase in opacity is observed with film paint thickness, reaching near complete coverage (>98.5%) at 120 μm.

## Conclusions

Acrylic-starch hybrid latexes with a high final biomaterial content were produced by seeded semibatch emulsion polymerization. To this end, fully biobased starch-zein bioparticles were incorporated as seeds in the emulsion polymerization, and three bioderived monomers, IBOMA, 2OA, and 2OMA, were used to replace petroleum-based monomers such as MMA and BA.

In this way, stable hybrid latexes with 20% and 32% solids and biomaterial content ranging from 70 to 78% were successfully synthesized. These hybrid latexes, with acrylic *T*_g_ values ranging from −5 to 11 °C depending on the biomonomer formulation, can form homogeneous and translucent films with an MFFT below −1 °C.

The morphology of the hybrid films consists of a continuous but not fully coalesced acrylic phase due to the presence of both large and small hard BP domains. This film morphology is consistent with the main particle populations of the latex, which involve: (i) multilobular hybrid particles with a BPs core surrounded by acrylic polymer lobes, produced by seed-mediated polymerization; (ii) pure acrylic particles formed by homogeneous or micellar nucleation; and (iii) free small BPs.

The continuous acrylic phase in the hybrid promotes rapid dissolution in an organic solvent like MEK, while simultaneously limiting film water uptake, resulting in swelling degrees below 50% across all samples.

The incorporation of hard domains of BPs generates a reinforcement effect in hybrid films, which exhibit Young’s modulus and tensile strength values ranging from 13 to 66 MPa and 1.6 to 4.9 MPa, respectively. In addition, higher values for both properties are obtained in LS latexes associated with greater BPs content.

Finally, as proof of concept, a hybrid latex with high HS content was used as a binder to formulate a white water-based paint, resulting in a stable system with 23% PVC and 35% solids content. The paint exhibits good coverage at wet film thicknesses of 120 μm or more, a matte finish, and a highly hydrophobic surface (contact angle = 98°) but low resistance to water exposure. Although water resistance remains a key property to be improved in these hybrid binders, the substantial enhancement in sustainability offered by these biobased coatings is noteworthy.

## Supplementary Material



## Notes

Characteristics of biobased monomers, evolution of the number of polymer particles of the LS and HS experiments, DSC thermograms of the obtained polymers, and TEM images of representative hybrid films made from HS latexes and mechanical properties of the hybrid films (PDF)
