# Supplementary material for: Sustainable Hybrid Latexes Derived from Starch Bioparticles and Biobased Monomers
Source: Biomacromolecules. 2025 Aug 29;26(10):7002–12. doi: 10.1021/acs.biomac.5c01315 (PMC12522144; doi:10.1021/acs.biomac.5c01315)
Supplement: Supplementary file 1 [file bm5c01315_si_001.pdf]

Supporting Information

Sustainable hybrid latexes derived from starch bioparticles and bio-based monomers

Sofía F. Cabrera[1], Aitor Barquero [2], Ludmila I. Ronco[1,3], Sara Beldarrain[2], Luis M. Gugliotta[1,3], Roque J. Minari[1,3*], Jose R. Leiza[2*]

[1]Polymer Reaction Engineering Group, INTEC, Santa Fe, 3000, Argentina

[2] POLYMAT, Kimika Aplikatua Saila, Kimika Falkultatea, University of the Basque Country UPV/EHU, Donostia/San Sebastian 20018, España

[3]Facultad de Ingeniería Química, Universidad Nacional del Litoral, Santa Fe, 3000, Argentina

*Corresponding authors: rjminari@santafe-conicet.gov.ar, jrleiza@ehu.eus

Table S1. Bio-content of bio-based monomers, their origin, and T_g of corresponding homopolymers.

Bio-based monomer	Bio [%][a]	*Origin*	*T_g homopolymer [°C]*
**IBOMA**	71	Pine resin	150
**2OA**	73	Castor oil	- 44
**2OMA**	67	Castor oil	0

[a] Weight percent of renewable content in the molecular structure.

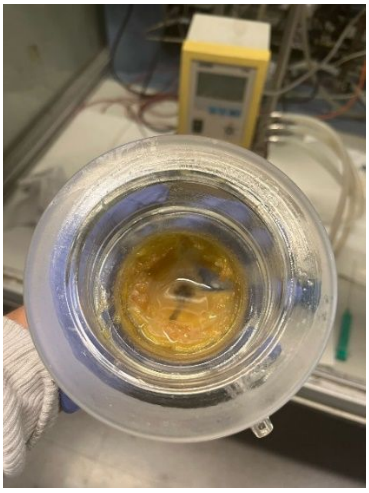

Figure S1. Picture showing the destabilized latex obtained with 32% solid content using a BPs/monomer mass ratio of 25/100 and a monomeric formulation of IBOMA/2OA 35/65.

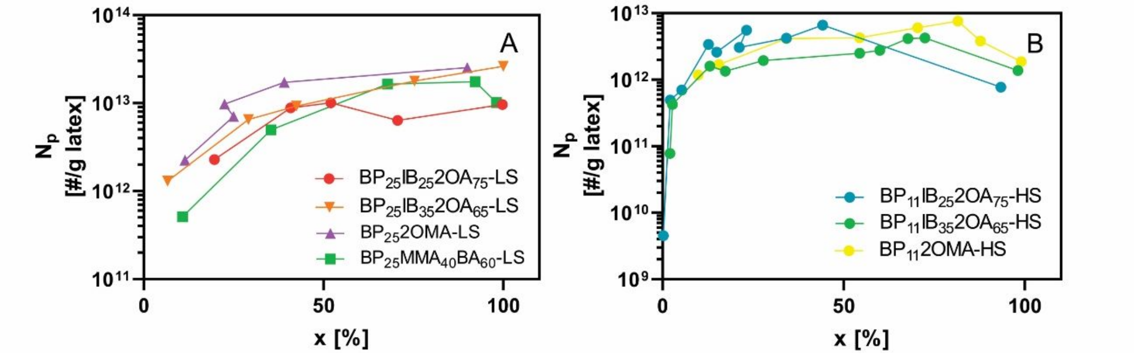

Figure S2. Evolution of the number of polymer particles along monomer conversion for latexes produced with LS (A) and HS (B).

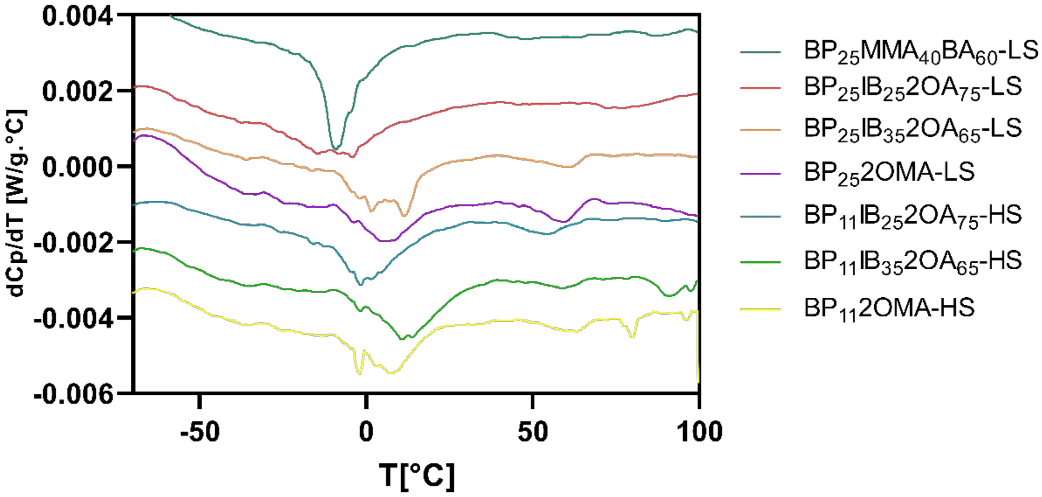

Figure S3. Derivative DSC thermograms for the different synthesized hybrid latexes.

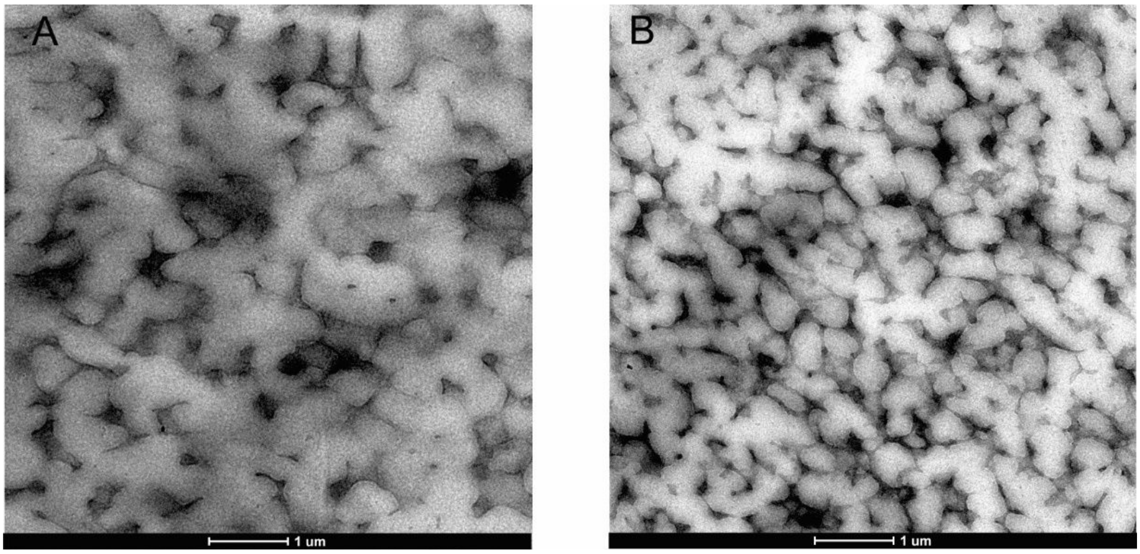

Figure S4. TEM images from transversal cuts of hybrid films formed from LS latexes $BP_{25}IB_{25}2OA_{75}$-LS and $BP_{25}IB_{35}2OA_{65}$-LS.

Table S2. Tensile properties of bio-based films obtained from LS latexes and the reference petroleum-based film.

Film	Young module (MPa)	Elongación at break (%)	Tensile strength (MPa)	Toughness (MPa)
$BP_{25}IB_{25}2OA_{75}$-LS	25.5 ± 4.6	372.3 ± 60.8	2.2 ± 0.1	7.01 ± 0.76
$BP_{25}IB_{35}2OA_{65}$-LS	65.1 ± 4.8	190.4 ± 6.0	4.9 ± 0.1	7.32 ± 0.19
BP_{25}2OMA-LS	66.6 ± 25.4	212.7 ± 27.6	4.6 ± 0.3	8.42 ± 0.67
$BP_{25}MMA_{40}BA_{60}$-LS*	37.7 ± 3.2	239.5 ± 13.9	3.75 ± 0.1	7.83 ± 0.13

* hybrid film produced with petroleum based acrylic monomers

Table S3. Tensile properties of bio-based films obtained from HS latexes.

Film	Young module (MPa)	Elongación at break (%)	Tensile strength (MPa)	Toughness (MPa)
$BP_{11}IB_{25}2OA_{75}$-HS	13.3 ± 1.5	1391.4 ± 33.0	1.63 ± 0.1	17.4 ± 0.9
$BP_{11}IB_{35}2OA_{65}$-HS	60.9 ± 9.2	572.2 ± 58.2	3.6 ± 0.3	15.9 ± 2.9
BP_{11}2OMA-HS	17.2 ± 3.2	1261.7 ± 99.8	2.9 ± 0.1	21.9 ± 2.2
